# Enzyme kinetics of dUTPase from the planarian *Dugesia ryukyuensis*

**DOI:** 10.1186/s13104-019-4191-6

**Published:** 2019-03-22

**Authors:** Md. Shahanoor Alam, Hideaki Moriyama, Midori Matsumoto

**Affiliations:** 1 Department of Bioscience and Informatics, Keio University, 3-14-1 Hiyoshi, Kouhoku-ku, Yokohama, 223-8522 Japan; 2 School of Biological Sciences, University of Nebraska-Lincoln, 243 Manter Hall, Lincoln, NE 68588 USA

**Keywords:** dUTPase, Planarian, Regeneration

## Abstract

**Objective:**

Planarians including *Dugesia ryukyuensis* (Dr) have strong regenerative abilities that require enhanced DNA replication. Knockdown of the DUT gene in Dr, which encodes deoxyuridine 5′-triphosphate pyrophosphatase (dUTPase), promotes DNA fragmentation, inhibits regeneration, and eventually leads to death. dUTPase catalyzes the hydrolysis of dUTP to dUMP and pyrophosphate. dUTPase is known to prevent uracil misincorporation in DNA by balancing the intracellular ratio between dUTP and dTTP, and contributes to genome stability. Nevertheless, the catalytic performance of Dr-dUTPase has not been reported.

**Results:**

To confirm the catalytic activity of Dr-dUTPase, we cloned and expressed Dr-DUT in *E. coli*. Then, we purified Dr-dUTPase using His-tag and removed the tag with thrombin. The resulting Dr-dUTPase had the leading peptide Gly–Ser–His– originating from the vector at the amino terminus, and a mutation, Arg66Lys, to remove the internal thrombin site. We observed the hydrolysis of dUTP by Dr-dUTPase using Cresol Red as a proton sensor. The *K*_*m*_ for dUTP was determined to be 4.0 µM, which is similar to that for human dUTPase. Dr-dUTPase exhibited a preference for dUTP over the other nucleotides. We conclude the Dr-dUTPase has catalytic activity.

**Electronic supplementary material:**

The online version of this article (10.1186/s13104-019-4191-6) contains supplementary material, which is available to authorized users.

## Introduction

The planarian *Dugesia ryukyuensis* is a model system for studying reproductive strategies for survival [1–3]. We identified key genes in its sexualization and regeneration, including a DNA repair gene, Dr-Rad51 [4], and a germ cell regulator gene, Dr-nanos [5]. Since these genes are also involved in genome stability, we hypothesized that the Dr-dut gene involved in nucleoside triphosphate biosynthesis [6] affects Dr-Rad51. The results of Dr-dut knockdown supported this hypothesis, upon which we observed alteration of the expression levels of Dr-rad51, Dr-rad51c, and the DNA damage response gene Dr-atm [7]. Nevertheless, the catalytic performance of Dr-dUTPase has not been reported.

The DUT gene encodes the enzyme deoxyuridine 5′-triphosphate pyrophosphatase (dUTPase; EC 3.6.1.23) [8]. Human (*Homo sapiens*, Hs) dUTPase is one of the most intensively studied enzymes [9] and is involved in pyrimidine metabolism. Specifically, Hs-dUTPase provides dUMP to thymidylate synthase [10] in the pathway of thymidine nucleotide biosynthesis [11]. By hydrolyzing dUTP, dUTPase decreases the intracellular concentration of dUTP, and hence lowers the probability of uracil being incorporated into DNA [9, 12]. In the biomedical field, Hs-dUTPase has been identified as a new target of cancer chemotherapy, and a dUTPase inhibitor (TAS-114) is under clinical study [13, 14].

**Fig. 1:**
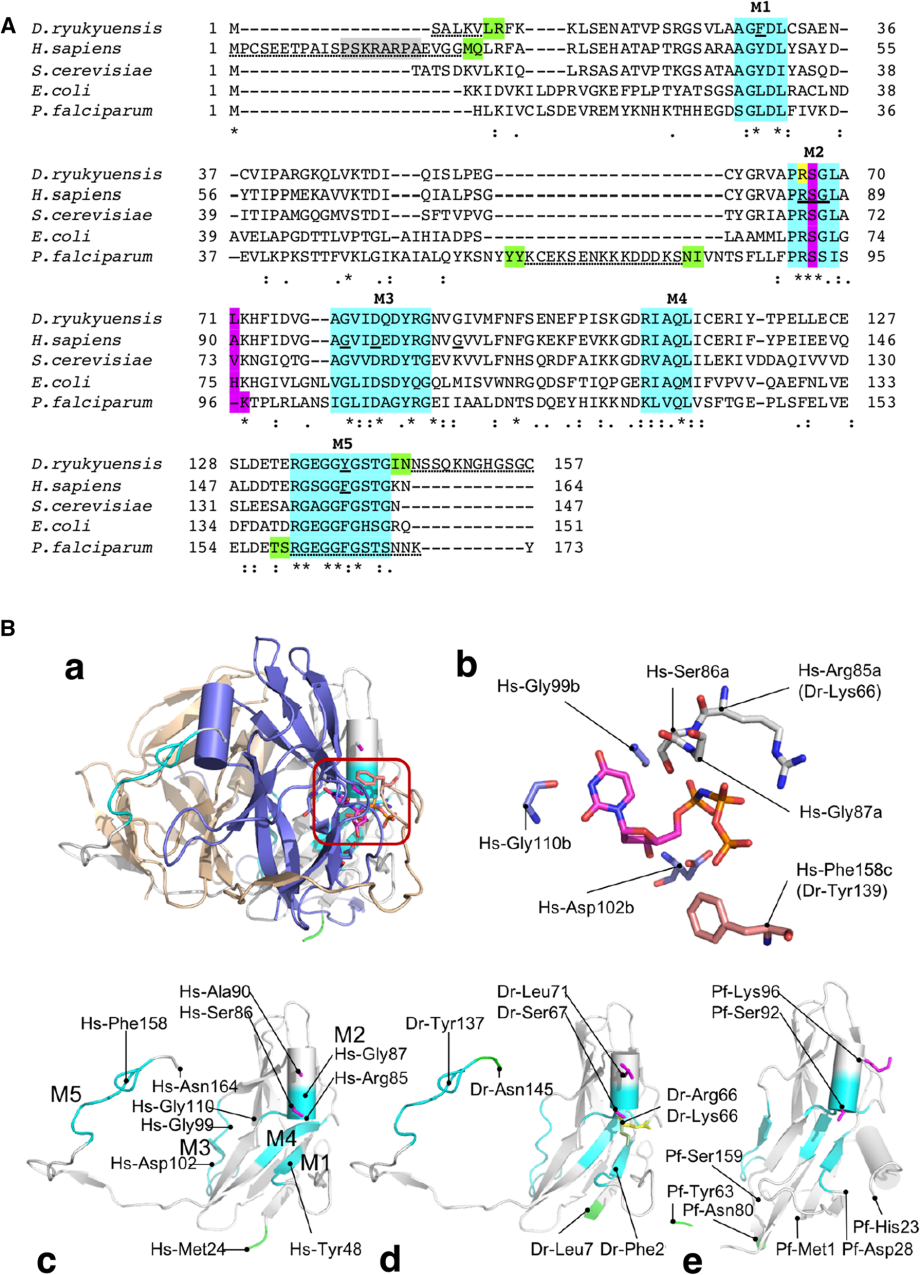
Structures of dUTPases. **A** Multiple amino acid sequence alignment of dUTPases. Sequences were presented with the similarity symbols, matches (*), highly similar (:), and medium similarity (.). The conserved motifs, M1–M5, were highlighted cyan. Sequences in α-helix in M2 involved in the initial grasp of substrate-water complex were highlighted magenta [19]. In *D. ryukyuensis* dUTPase, an intentional mutation in the expression construct, Arg66Lys, to avoid thrombin cleavage was shown in yellow (Additional file 1: Fig. S1), and potential compensatory substitutions against *H. sapiens* dUTPase, Phe29 and Tyr139, were indicated by underline. In *H. sapiens* dUTPase, reported nuclear localization signal [29] was highlighted gray, and residues involved in uracil recognition in the conserved motif [9] were indicated by underlines. In dUTPases from *D. ryukyuensis*, *H. sapiens* and *P. falciparum*, missing amino acid residues in the 3D-structure models were marked by doted underlines, and two neighboring residues were marked green as visual markers (**B**). **B** 3D structure of dUTPase. **a** A trimer view of Hs-dUTPase in the cartoon view (PDB code, 3ehw) with only the residues involved in the active site centered the α–helix of A chain were shown in sticks (magenta enclosing). **b** A schematic diagram of substrate, an inhibitor dUMPNPP (2′-deoxyuridine-5′-[(α,β)-imido]triphosphate), and active site residues from the enclosing in **A**. **c** A monomer view of Hs-dUTPase. **d** A monomer view of Dr-dUTPase model. **e** A monomer view of Pf-dUTPase (1vyq). In c-e, color coding matched with **A**

Two major isoforms of Hs-dUTPase have been reported. Isoform 2 (164 amino acids; Fig. 1A) is localized in the nucleus, while isoform 3 (252 amino acids) is localized in the mitochondria [15, 16]. The 3D structure of isoform 2 was solved, revealing that the enzyme adopts a homo-trimer form [17]. However, in Dr-dUTPase, RNA sequencing revealed only one form of dUTPase [18].

Trimer dUTPases have three active sites at the subunit boundaries, consisting of five conserved motifs (Fig. 1A) [12, 19]. The *K*_*m*_ of Hs-dUTPase for dUTP was reported to be 1.1 µM [20, 21]. To confirm the catalytic activity of Dr-dUTPase, we cloned, expressed, and purified the enzyme, and then measured its activity.

## Main text

### Materials and methods

1. *Sequence analysis* Sequences of trimer dUTPases from *Dugesia ryukyuensis* (Dr; DDBJ ID, LC421836), *Homo sapiens* (Hs; UniProt ID, P33316-2), *Saccharomyces cerevisiae* (Sc; P33317), *Escherichia coli* (Ec; P06968), and *Plasmodium falciparum* (Pf; Q8II92) were used because of the availability of kinetic data and 3D structures. Sequences were aligned by MAFFT [22] using the L-INS-I method [23] and BLOSUM62 scoring matrix [24].
2. *Preparation of Dr*-*dUTPase* The deduced amino acid sequence of Dr-dUTPase contained an internal thrombin cleavage site at Arg66 (Fig. 1A). To remove the thrombin cleavage site, the *Dr*-*dut* gene was mutated from G to A at position 197. Codon utilization of Dr-DUT was optimized to the expression host *E. coli* (Codon Optimization Tool, https://www.idtdna.com/codonopt; Additional file 1: Fig. S1, Table S1) and synthesized (Integrated DNA Technologies, Coralville, IA, USA). *Dr*-*dut* was cloned into the pET-15b vector (Novagen, San Diego, CA, USA) using *Nde*I and *Bam*HI sites. Dr-dUTPase was prepared as described previously [19]. Briefly, *E. coli* BL21 (DE3) was transformed by the construct and cultivated in LB medium. Expression of the *Dr*-*dut* gene was induced by IPTG. His-tagged Dr-dUTPase was purified using a Ni–NTA column (GE Healthcare, Chicago, IL, USA). Produced Dr-dUTPase contained the leading peptide MGSSHHHHHHSSGLVPRGSH at the amino terminus. The purified fraction (Additional file 1: Fig. S2) was subjected to desalting by dialysis against 1× thrombin buffer (150 mM NaCl, 1.5 mM CaCl_2_, 20 mM Tris–HCl, pH 8.0) before the thrombin cleavage. After the thrombin cleavage, the resulting Dr-dUTPase still contained the leading peptide GSH at the amino terminus.
3. *Kinetic measurements* The enzymatic activity was monitored by the color change of Cresol Red due to the production of protons by the hydrolysis of nucleotides [19] using a DU-640 UV/Vis spectrophotometer (Beckman Coulter, Brea, CA, USA). A total of 990 µl of base buffer (100 mM KCl, 5 mM MgCl_2_, 0.25 mM Bicine, pH 8.2) containing final concentrations of 10 µM dUTP + 25 µM Cresol Red was injected into a cuvette (optical path of 1 cm) containing 10 µl of 0.02 mM enzyme in the base buffer. Absorbance at 573 nm was recorded at intervals of 1.1 s (Additional file 1: Fig. S3). The recorded color change as a function of time was converted to the reaction product, and the *K*_*m*_ values were calculated by the integrated Michaelis–Menten method, as described by Larsson et al. [20] and Inoguchi et al. [19].
4. *Structural bioinformatics* The 3D structures of dUTPase from Hs (PDB ID, 3ehw) and Pf (1vyq) were used. Homology modeling of intact Dr-dUTPase was performed on the SWISS-MODEL server [25]. The system ranked Hs-dUTPase (3ehw) as the template at the top, and the established model obtained a QMEAN value of − 0.21 with the residue range between 7 and 145. The root-mean-square distance (RMSD) between the template and model was 0.1 Å in both monomer and trimer forms. Taking these findings together, the model of *D. ryukyuensis* dUTPase was judged to be usable for structural mining. Modeling for Dr-dUTPase Arg66Lys resulted in the same folding as with the native Dr-dUTPase, with RMSD against Hs-dUTPase in trimer of 0.1 Å. Structural mining and graphics preparation were performed using the PyMOL Molecular Graphics System, Version v1.7.6.3 (Schrödinger, LLC, New York City, NY, USA).

### Results

1. *Amino acid sequence of Dr*-*dUTPase.* The planarian *Dr*-*dut* gene [7] contained an ORF of 462 bp encoding a polypeptide of 153 amino acid residues (Fig. 1A; Additional file 1: Fig. S1). The deduced molecular mass and pI of Dr-dUTPase were 16,554 and 5.7, respectively. The amino acid sequence alignment revealed that Dr-dUTPase possesses the five conserved motifs unique to homotrimeric dUTPases, including Hs-dUTPase [12]. Dr-dUTPase and Hs-dUTPase share sequence identity of 70%.
2. *Production of Dr*-*dUTPase* After the *Dr*-*dut* gene had been mutated to exclude the internal thrombin site, its codon utilization was optimized to *E. coli* (Additional file 1: Fig. S1). The mutation Arg66Lys in motif 2 was chosen to retain the positive charge of the side chain. The pI of mutated dUTPase was thus predicted to remain at 5.7. Alteration of the enzyme may be unavoidable because the residue is located next to Ser67, which is a key residue for the active site. The optimization gave evenly distributed usage of codon utilization (Additional file 1: Table S1), and increased the GC content of the *Dr*-*dut* gene from 37 to 50%. The genomic GC content of *E. coli* was reported to be 50% [26]. The gene was cloned into the pET-15 vector and expressed in *E. coli*. After affinity purification (Additional file 1: Fig. S2A), the typical yield was about 20 mg of purified tagged protein from 250 ml of culture. Thrombin cleavage was performed to remove the His-tag. The resulting Dr-dUTPase contained the leading GSH peptide and was 156 amino acids in length. The deduced mass and pI were 16,807 and 5.9, respectively. The enzyme was purified uniformly showing the single band in the SDS-PAGE (Additional file 1: Fig. S2B). In the concentration by centrifugation using the 30 kDa-cut membrane (YM-30, MilliporeSigma, Burlington, MA, USA), Dr-dUTPase was retained. Therefore, Dr-dUTPase was potentially existed in oligomer form, including the trimer.
3. *Enzymatic activity of Dr*-*dUTPase* To assess the catalytic activity of Dr-dUTPase, hydrolysis of dUTP was monitored by the color change of Cresol Red. The change in absorbance upon multiple-turnover hydrolysis of dUTP as a function of time was converted to the amount of product at a certain concentration of substrate (Additional file 1: Fig. S3) [20]. To obtain *K*_*m*_ and *V*_*max*_, the reaction rate against the substrate concentration was fitted to the integrated Michaelis–Menten equation (Additional file 1: Fig. S3 inset). Dr-dUTPase exhibits enzymatic activity, with estimated *K*_*m*_ and *V*_*max*_ values of 4.0 μM and 20.2 μM s^−1^, respectively, at pH 8.2 and 25 °C (Table 1). *k*_*ca*t_ was estimated to be 3.4 × 10 s^−1^, assuming three active sites per trimer of dUTPase. The specificity constant, *k*_*cat*_/*K*_*m*_, for dUTP was thus 8.5 × 10^6^ M^−1^ s^−1^. Dr-dUTPase exhibited catalytic activity for dUTP.

**Table 1:** Catalytic parameters and specificity constant of *Dr*-dUTPase for different nucleotides

Nucleotides	*K*_*m*_ (μM)	*k*_*cat*_ (s^−1^)	*k*_*cat*_*/K*_*m*_ (M^−1^ s^−1^)
dUTP (n = 7)	4.0 ± 0.4	3.4 × 10 ± 3.0	8.5 × 10^6^ ± 1.1 × 10^6^
dATP	9.4 × 10 ± 1.2	1.5 ± 0.2	1.6 × 10^4^ ± 2.1 × 10^3^
dTTP	1.1 × 10^2^ ± 7.2	1.4 ± 0.3	1.3 × 10^4^ ± 2.8 × 10^3^
dCTP	7.7 × 10 ± 3.5	1.6 ± 0.2	2.1 × 10^4^ ± 2.7 × 10^3^
dGTP	2.1 × 10^2^ ± 1.0 × 10	2.9 ± 0.5	1.4 × 10^4^ ± 2.4 × 10^3^
dUTP, 5 mM MnCl_2_	7.3 ± 1.2	4.8 × 10 ± 6.8	6.5 × 10^6^ ± 2.1 × 10^6^

Measurements were triplicated (n = 3) except in the case of dUTP with MgCl_2_

To analyze the substrate specificity of Dr-dUTPase, enzyme assays were performed using dATP, dTTP, dCTP, and dGTP as substrates at a final concentration of 100 µM and 4–6 µM dUTPase (Table 1). The *K*_*m*_ obtained for the tested nucleotides ranged between 94 and 210 (Table 1). However, the performance of Dr-dUTPase for nucleotides other than dUTP was 2 orders of magnitude lower. Dr-dUTPase in the present format prefers dUTP as its substrate, while allowing the hydrolysis of dATP, dTTP, dCTP, and dGTP at lower levels.

To test the metal dependence of Dr-dUTPase, an enzyme assay was performed after the dialysis, without adding Mg^2+^. In the dialyzed enzyme, Dr-dUTPase activity was not detected, but the activity was regained as Mg^2+^ was added to the system. However, the *K*_*m*_ value remained unchanged at MgCl_2_ concentrations from 5 to 25 mM. When the magnesium was replaced with 5 mM MnCl_2_, Dr-dUTPase maintained its activity (Table 1). When magnesium was replaced with 5 mM CaCl_2_, enzymatic activity was not observed. These results suggest that the activity of Dr-dUTPase occurs in a manner dependent on the metal ions magnesium and manganese.

### Discussion

Feeding based knockdown of *Dr*-*dut* invited planarian death. To obtain further knowledge about the mechanism of the cell death, we analyzed the catalytic performance of Dr-dUTPase.

Dr-dUTPase exhibited catalytic activity, as other trimer dUTPases do (Table 2). Interestingly, its tolerance for other nucleotides was unexpectedly high. For instance, the difference in the specificity constant between dUTP and other nucleotides, and Dr-dUTPase and other dUTPases were 100-fold and about 1000-fold, respectively (Table 2).

**Table 2:** Michaelis–Menten constant, *K*_*m*_ (μM), and specificity constant, *k*_*cat*_*/K*_*m*_ (M^−1^ s^−1^) of dUTPases from different origins

Substrate	*D. ryukyuensis*	*H. sapiens*	*S. cerevisiae*	*E. coli*	*P. falciparum*
dUTP	4.0 (8.5 × 10^6^)	1.1 (6.6 × 10^6^)	1.3 × 10^2^ (7.4 × 10^5^)	0.2 (3.6 × 10^6^)	1.9 (7.1 × 10^6^)
dATP	9.4 × 10 (1.6 × 10^4^)	NR	NR	NR	1.1 × 10^3^ (2.7)
dTTP	1.1 × 10^2^ (1.3 × 10^4^)	7.6 × 10^2^ (2.0 × 10)	NR	> 2.0 × 10^4^ (< 0.1 × 10^3^)	2.9 × 10^2^ (9.2)
dCTP	7.7 × 10 (2.1 × 10^4^)	6.9 × 10^2^ (1.6 × 10^2^)	NR	4.0 × 10^3^ (0.1 × 10^3^)	4.7 × 10^2^ (2.2 × 10)
dGTP	2.1 × 10^2^ (1.4 × 10^4^)	NR	NR	NR	2.0 × 10^3^ (1.3 × 10^−1^)
Sources	This study	[21]	[28]	[20]	[21]

In dUTPase from *E. coli*, the *K*_*m*_ for dTTP and dCTP were presented congruent to *K*_*d*_

*NR* not reported

Potential causes of the differences in biochemistry included the difference in the amino acid sequence in key residues in conserved motifs (M1-5; Fig. 1A). In the case of Hs-dUTPase, the active site for the A chain consists of Arg85, Ser86, and Gly87; for the B chain, Gly99, Asp102, and Gly110; and for the C chain Phe158 [9] (Fig. 1A, B). In the present form of Dr-dUTPase, substitutions were found as follows: Arg85Lys in the A chain and Phe158Tyr in the C chain. Among them, Phe158 in Hs-dUTPase was suggested to promote stacking enabling uracil recognition [27]. Phe158 in Hs-dUTPase is conserved in other dUTPases, including Sc-dUTPase, Ec-dUTPase, and Pf-dUTPase. By the substitution of Phe158Tyr, Dr-dUTPase potentially obtained alternative stacking between the aromatic ring in the residue and the uracil base. The alternative staking could have allowed the larger ligand tolerance.

In motif 2 of trimer dUTPase, there is only one short α-helix, which holds two amino acid facing active site (Fig. 1A, B). One is the nucleophile Ser, and another is the four residues after it. Combination of the amino acid between the Ser and another residue seemed affect the affinity of the enzyme in terms of the initial capture of the substrate–water complex [19]. In hs-dUTPase and Sc-dUTPase have small hydrophobic residues, and they show a lower affinity then that of Ec-dUTPase which carry the positively charged residues at the corresponding site. In Dr-dUTPase has Ser and Leu on the α-helix. Our results of the activity assay showed Dr-dUTPase has lower affinity to dUTP than that of Hs-dUTPase (Table 2). Nevertheless, Pf-dUTPase had positively charge amino acid at the second position in the helix, this enzyme had a large insertion in the leading sequence of M2. Hence Pf-dUTPase is an exception case.

The present study shows that Dr-dUTPase has catalytic activity that preferentially favors dUTP, but also possesses high tolerance regarding substrate recognition. This result for Dr-dUTPase was unexpected because Hs-dUTPase can slightly recognize dTTP and dCTP, but not dATP and dGTP (Table 2). If this is also the case in vivo, the wide substrate acceptance of Dr-dUTPase would be notable as a planarian-specific phenomenon. Either way, the results of the present study confirm the catalytic activity of dUTPase, increasing our basic understanding of the roles of dUTPase in planarians.

## Limitations

Dr-dUTPase has conserved motif of trimer dUTPase (M1-5), and has conserved residues for the inter-subunit interactions. Then we assumed that Dr-dUTPase keeps a trimer structure. To conform the oligomer state of Dr-dUTPase, we performed the size exclusion chromatography, but unfortunately it could not succeed.

The 3D structures of dUTPase showed that Dr-Tyr139 (B-chain) and Hs-Phe158 (B-chain) in motif 5 (M5), are involved in stacking between the U-base and aromatic ring of the side chain. The Dr-Phe29 (C-chain) and Hs-Tyr48 (C-chain) are located in structural motif 1 (M1), which is a part of the active sites. The aspartate in M1, next to the Dr-Phe29 (C-chain) and Hs-Tyr48, interacts with active site water molecules to stabilize the Mg ion. Therefore, Dr-Phe29 (C-chain) and Hs-Tyr48 are still involved in the substrate binding. Nevertheless, we do not have the crystal structure yet to support this idea.

## Additional file

**Additional file 1: Fig. S1.** Nucleotide sequence of Dr-DUT gene. Shown sequences are RNA sequencing (Rseq), mutated (G197A), and codon optimized for* E. coli*. **Fig. S2.** Production of Dr-dUTPase analyzed by SDS-PAGE. **Fig. S3.** Hydrolysis of dUTP by Dr-dUTPase. **Table S1.** Codon utilization in Dr-DUT Rseq and COTEC.

## Abbreviations

dUTPase: deoxyuridine 5′-triphosphate pyrophosphatase
RMSD: root-mean-square distance

## References

[1] Kawakatsu M, Oki I, Tamura S (1995). Taxonomy and geographical-distribution of Dugesia-Japonica and *D ryukyuensis* in the Far-East. Hydrobiologia.

[2] Hoshi M, Kobayashi K, Arioka S, Hase S, Matsumoto M (2003). Switch from asexual to sexual reproduction in the planarian *Dugesia ryukyuensis*. Integr Comp Biol.

[3] Chinone A, Nodono H, Matsumoto M (2014). Triploid planarian reproduces truly bisexually with euploid gametes produced through a different meiotic system between sex. Chromosoma.

[4] Chinone A, Matsumoto M (2014). DrRad51 is required for chiasmata formation in meiosis in planarian *Dugesia ryukyuensis*. Mol Reprod Dev.

[5] Nakagawa H, Ishizu H, Chinone A, Kobayashi K, Matsumoto M (2012). The Dr-nanos gene is essential for germ cell specification in the planarian *Dugesia ryukyuensis*. Int J Dev Biol.

[6] Hirmondo R, Lopata A, Suranyi EV, Vertessy BG, Toth J (2017). Differential control of dNTP biosynthesis and genome integrity maintenance by the dUTPase superfamily enzymes. Sci Rep.

[7] Alam MS, Moriyama H, Matsumoto M (2018). Inhibition of *Dr*-*dut* gene causes DNA damage in planarian. Mol Reprod Dev.

[8] Bertani LE, Haggmark A, Reichard P (1961). Synthesis of pyrimidine deoxyribonucleoside diphosphates with enzymes from *Escherichia coli*. J Biol Chem.

[9] Mol CD, Harris JM, McIntosh EM, Tainer JA (1996). Human dUTP pyrophosphatase: uracil recognition by a beta hairpin and active sites formed by three separate subunits. Structure.

[10] Anderson DD, Quintero CM, Stover PJ (2011). Identification of a de novo thymidylate biosynthesis pathway in mammalian mitochondria. Proc Natl Acad Sci USA.

[11] Hardy LW, Finer-Moore JS, Montfort WR, Jones MO, Santi DV, Stroud RM (1987). Atomic structure of thymidylate synthase: target for rational drug design. Science.

[12] Vertessy BG, Toth J (2009). Keeping uracil out of DNA: physiological role, structure and catalytic mechanism of dUTPases. Acc Chem Res.

[13] Saito K, Nagashima H, Noguchi K, Yoshisue K, Yokogawa T, Matsushima E, Tahara T, Takagi S (2014). First-in-human, phase I dose-escalation study of single and multiple doses of a first-in-class enhancer of fluoropyrimidines, a dUTPase inhibitor (TAS-114) in healthy male volunteers. Cancer Chemother Pharmacol.

[14] Yano W, Yokogawa T, Wakasa T, Yamamura K, Fujioka A, Yoshisue K, Matsushima E, Miyahara S, Miyakoshi H, Taguchi J (2018). TAS-114, a first-in-class dual dUTPase/DPD inhibitor, demonstrates potential to improve therapeutic efficacy of fluoropyrimidine-based chemotherapy. Mol Cancer Ther.

[15] Ladner RD, McNulty DE, Carr SA, Roberts GD, Caradonna SJ (1996). Characterization of distinct nuclear and mitochondrial forms of human deoxyuridine triphosphate nucleotidohydrolase. J Biol Chem.

[16] Ladner RD, Carr SA, Huddleston MJ, McNulty DE, Caradonna SJ (1996). Identification of a consensus cyclin-dependent kinase phosphorylation site unique to the nuclear form of human deoxyuridine triphosphate nucleotidohydrolase. J Biol Chem.

[17] Varga B, Barabas O, Kovari J, Toth J, Hunyadi-Gulyas E, Klement E, Medzihradszky KF, Tolgyesi F, Fidy J, Vertessy BG (2007). Active site closure facilitates juxtaposition of reactant atoms for initiation of catalysis by human dUTPase. FEBS Lett.

[18] Ishizuka H, Maezawa T, Kawauchi J, Nodono H, Hirao Y, Nishimura O, Nakagawa H, Sekii K, Tasaka K, Tarui H (2007). The *Dugesia ryukyuensis* database as a molecular resource for studying switching of the reproductive system. Zool Sci.

[19] Inoguchi N, Chaiseeda K, Yamanishi M, Kim MK, Jang Y, Bajaj M, Chia CP, Becker DF, Moriyama H (2015). Structural insights into the mechanism defining substrate affinity in Arabidopsis thaliana dUTPase: the role of tryptophan 93 in ligand orientation. BMC Res Notes.

[20] Larsson G, Nyman PO, Kvassman JO (1996). Kinetic characterization of dUTPase from *Escherichia coli*. J Biol Chem.

[21] Quesada-Soriano I, Casas-Solvas JM, Recio E, Ruiz-Perez LM, Vargas-Berenguel A, Gonzalez-Pacanowska D, Garcia-Fuentes L (2010). Kinetic properties and specificity of trimeric *Plasmodium falciparum* and human dUTPases. Biochimie.

[22] Katoh K, Rozewicki J, Yamada KD (2017). MAFFT online service: multiple sequence alignment, interactive sequence choice and visualization. Brief Bioinform.

[23] Katoh K, Kuma K, Toh H, Miyata T (2005). MAFFT version 5: improvement in accuracy of multiple sequence alignment. Nucleic Acids Res.

[24] Henikoff S, Henikoff JF (1992). Amino acid substitution matrices from protein blocks. Proc Natl Acad Sci USA.

[25] Waterhouse A, Bertoni M, Bienert S, Studer G, Tauriello G, Gumienny R, Heer FT, de Beer TAP, Rempfer C, Bordoli L (2018). SWISS-MODEL: homology modelling of protein structures and complexes. Nucleic Acids Res.

[26] Riley M, Abe T, Arnaud MB, Berlyn MK, Blattner FR, Chaudhuri RR, Glasner JD, Horiuchi T, Keseler IM, Kosuge T (2006). *Escherichia coli* K-12: a cooperatively developed annotation snapshot–2005. Nucleic Acids Res.

[27] Pecsi I, Leveles I, Harmat V, Vertessy BG, Toth J (2010). Aromatic stacking between nucleobase and enzyme promotes phosphate ester hydrolysis in dUTPase. Nucleic Acids Res.

[28] Tchigvintsev A, Singer AU, Flick R, Petit P, Brown G, Evdokimova E, Savchenko A, Yakunin AF (2011). Structure and activity of the *Saccharomyces cerevisiae* dUTP pyrophosphatase DUT1, an essential housekeeping enzyme. Biochem J.

[29] Bozoky Z, Rona G, Klement E, Medzihradszky KF, Merenyi G, Vertessy BG, Friedrich P (2011). Calpain-catalyzed proteolysis of human dUTPase specifically removes the nuclear localization signal peptide. PLoS ONE.

