# Supplementary material for: Enzyme kinetics of dUTPase from the planarian Dugesia ryukyuensis
Source: BMC Res Notes. 2019 Mar 22;12:163. doi: 10.1186/s13104-019-4191-6 (PMC6431053; doi:10.1186/s13104-019-4191-6)
Supplement: Supplementary file 1 — Additional file 1: Fig. S1. Nucleotide sequence of Dr-DUT gene. Shown sequences are RNA sequencing (Rseq), mutated (G197A), and codon optimized for E. coli. Fig. S2. Production of Dr-dUTPase analyzed by SDS-PAGE. Fig. S3. Hydrolysis of dUTP by Dr-dUTPase. Table S1. Codon utilization in Dr-DUT Rseq and COTEC. [file 13104_2019_4191_MOESM1_ESM.docx]

**Additional file 1:**

**Fig. S1 Nucleotide sequence of Dr-DUT gene.** Shown sequences are RNA sequencing (Rseq), mutated (G197A), and codon optimized for *E. coli*. Underlined codon, AGA encodes Arg, was mutated to AAA encodes Lys by the G197A substitution. Asterisks (*) and caps (^)

10 20 30 40 50 60

Rseq atgtctgctttgaaagtgttgagatttaagaaattatcagaaaacgcaactgttccatct

G197A atgtctgctttgaaagtgttgagatttaagaaattatcagaaaacgcaactgttccatct

COTEC atgtcagccttgaaagtgctgcggtttaagaaactgtcggagaatgccactgttccaagt

*****^**^*********^**^*^*********^*^**^**^**^**^*********^^*

M S A L K V L R F K K L S E N A T V P S

70 80 90 100 110 120

Rseq cggggttctgtcttagcagcaggttttgatttatgtagtgctgaaaattgtgttatccca

G197A cggggttctgtcttagcagcaggttttgatttatgtagtgctgaaaattgtgttatccca

COTEC cgtggcagcgtacttgcggctggttttgacctgtgtagcgcggaaaattgcgtgattccg

**^**^^^^**^^*^**^**^********^^*^*****^**^********^**^**^**^

R G S V L A A G F D L C S A E N C V I P

130 140 150 160 170 180

Rseq gccagaggcaagcaattagtaaagacggatatccagattagtcttcctgaaggatgttat

G197A gccagaggcaagcaattagtaaagacggatatccagattagtcttcctgaaggatgttat

COTEC gctcgtggcaaacaactggtgaaaaccgacattcagatctctttgcccgaaggttgctac

**^^*^*****^***^*^**^**^**^**^**^*****^^^*^*^**^*****^**^**^

A R G K Q L V K T D I Q I S L P E G C Y

190 200 210 220 230 240

Rseq ggaagagttgctccaagaagtggtttagcattaaaacattttattgatgtcggtgctggt

G197A ggaagagttgctccaaaaagtggtttagcattaaaacattttattgatgtcggtgctggt

COTEC ggtcgtgttgcacctaaatctggtttagccctgaaacacttcatcgatgtaggtgcagga

**^^*^*****^**^*^*^^*********^^*^*****^**^**^*****^*****^**^

G R V A P RK S G L A L K H F I D V G A G

250 260 270 280 290 300

Rseq gtcatagatcaagattatcgtggaaatgttgggattgtcatgttcaacttctcagaaaat

G197A gtcatagatcaagattatcgtggaaatgttgggattgtcatgttcaacttctcagaaaat

COTEC gtcattgaccaggattatcgcgggaatgtgggcatcgtcatgttcaactttagcgagaac

*****^**^**^********^**^*****^**^**^**************^^^^**^**^

E F P I S K G D R I A Q L I C E R I Y T

310 320 330 340 350 360

Rseq gaatttccaatttccaaaggagatagaattgctcaacttatttgtgaaagaatatataca

G197A gaatttccaatttccaaaggagatagaattgctcaacttatttgtgaaagaatatataca

COTEC gaattcccgatttccaaaggcgatcgcattgcgcaactgatttgcgaacgcatctacaca

*****^**^***********^***^*^*****^*****^*****^***^*^**^**^***

E F P I S K G D R I A Q L I C E R I Y T

370 380 390 400 410 420

Rseq cctgaattattagaatgtgaatccttggatgagacagaacgtggtgaaggtggttatgga

G185A cctgaattattagaatgtgaatccttggatgagacagaacgtggtgaaggtggttatgga

COTEC ccggagttactggagtgtgaaagcctcgatgaaaccgaacgcggcgaaggtggctatggc

**^**^***^*^**^******^^*^*^*****^**^*****^**^********^*****^

P E L L E C E S L D E T E R G E G G Y G

430 440 450 460 462

Rseq tctacaggaattaacaattcttctcagaaaaatggtcattag

G185A tctacaggaattaacaattcttctcagaaaaatggtcattag

COTEC agtacgggaatcaacaactcgtcccagaagaatgggcattaa

^^***^*****^*****^**^**^*****^*****^*****^

S T G I N N S S Q K N G H Stop

**Fig. S2 Production of Dr-dUTPase analyzed by SDS-PAGE.** (A) Production of His-tagged Dr-dUTPase. Lanes are: M, Molecular weight marker; 1, clear lysate after sonication of cell pellet; 2, flow-through fraction of affinity column chromatography; 3, flow-through during washing of column; 4-6, eluted His-tagged dUTPase at 0.2M imidazole. (B) Removal of the His-tag. Lanes are: M, Molecular weight marker; 1, Dr*-*dUTPase before thrombin cleavage, lane-2, Dr*-*dUTPase after thrombin cleavage to remove His-tag.

**Fig. S3 Hydrolysis of dUTP by Dr*-*dUTPase**. The reaction trace was recorded starting with the solution conditions, 10 µM dUTP and 0.02 µM dUTPase (Materials and method). The solid line in the figure shows the best-fit line used to estimate the *K_M_* value. The inset graph shows the linear regression to estimate the *K_M_* value.

**
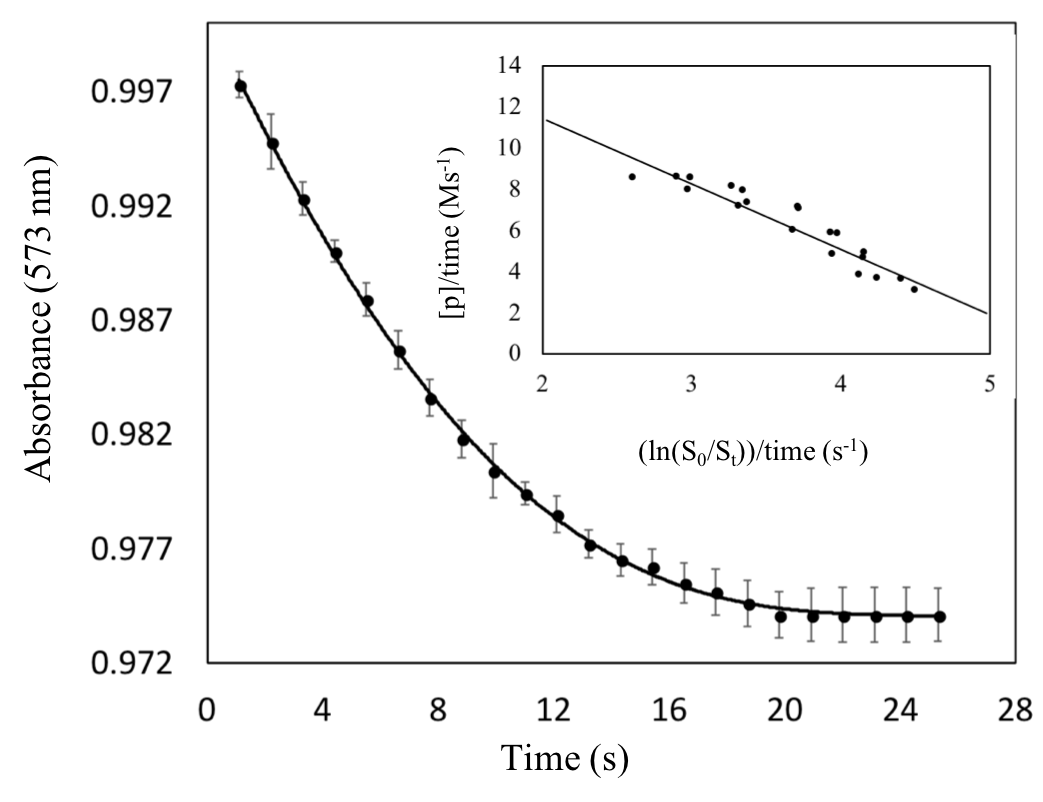
**

**Table S1. Codon utilization in Dr-DUT Rseq and COTEC**

| Amino acid | Codon | No. of codons in G197A | No. of codons in COTEC | Change in No. of codons | Codon fraction in  G197A | Codon fraction in  COTEC | Change in Codon fraction |
| --- | --- | --- | --- | --- | --- | --- | --- |
| Ala | GCG | 0 | 3 | -3 | 0 | 0.3 | -0.3 |
| Ala | GCA | 4 | 2 | 2 | 0.4 | 0.2 | 0.2 |
| Ala | GCT | 5 | 2 | 3 | 0.5 | 0.2 | 0.3 |
| Ala | GCC | 1 | 3 | -2 | 0.1 | 0.3 | -0.2 |
|  |  |  |  |  |  |  |  |
| Cys | TGT | 5 | 2 | 3 | 1 | 0.4 | 0.6 |
| Cys | TGC | 0 | 3 | -3 | 0 | 0.6 | -0.6 |
|  |  |  |  |  |  |  |  |
| Asp | GAT | 7 | 4 | 3 | 1 | 0.57 | 0.43 |
| Asp | GAC | 0 | 3 | -3 | 0 | 0.43 | -0.43 |
|  |  |  |  |  |  |  |  |
| Glu | GAG | 1 | 4 | -3 | 0.08 | 0.33 | -0.25 |
| Glu | GAA | 11 | 8 | 3 | 0.92 | 0.67 | 0.25 |
|  |  |  |  |  |  |  |  |
| Phe | TTT | 4 | 3 | 1 | 0.67 | 0.5 | 0.17 |
| Phe | TTC | 2 | 3 | -1 | 0.33 | 0.5 | -0.17 |
|  |  |  |  |  |  |  |  |
| Gly | GGG | 1 | 2 | -1 | 0.06 | 0.12 | -0.06 |
| Gly | GGA | 6 | 2 | 4 | 0.35 | 0.12 | 0.23 |
| Gly | GGT | 9 | 6 | 3 | 0.53 | 0.35 | 0.18 |
| Gly | GGC | 1 | 7 | -6 | 0.06 | 0.41 | -0.35 |
|  |  |  |  |  |  |  | 0 |
| His | CAT | 2 | 1 | 1 | 1 | 0.5 | 0.5 |
| His | CAC | 0 | 1 | -1 | 0 | 0.5 | -0.5 |
|  |  |  |  |  |  |  |  |
| Ile | ATA | 2 | 0 | 2 | 0.18 | 0 | 0.18 |
| Ile | ATT | 7 | 6 | 1 | 0.64 | 0.55 | 0.09 |
| Ile | ATC | 2 | 5 | -3 | 0.18 | 0.45 | -0.27 |
|  |  |  |  |  |  |  |  |
| Lys | AAG | 3 | 2 | 1 | 0.33 | 0.22 | 0.11 |
| Lys | AAA | 6 | 7 | -1 | 0.67 | 0.78 | -0.11 |
|  |  |  |  |  |  |  |  |
| Leu | TTG | 3 | 2 | 1 | 0.23 | 0.15 | 0.08 |
| Leu | TTA | 8 | 2 | 6 | 0.62 | 0.15 | 0.47 |
| Leu | CTG | 0 | 7 | -7 | 0 | 0.54 | -0.54 |
| Leu | CTA | 0 | 0 | 0 | 0 | 0 | 0 |
| Leu | CTT | 2 | 1 | 1 | 0.15 | 0.08 | 0.07 |
| Leu | CTC | 0 | 1 | -1 | 0 | 0.08 | -0.08 |
|  |  |  |  |  |  |  |  |
| Met | ATG | 2 | 2 | 0 | 1 | 1 | 0 |
|  |  |  |  |  |  |  |  |
| Asn | AAT | 5 | 4 | 1 | 0.63 | 0.5 | 0.13 |
| Asn | AAC | 3 | 4 | -1 | 0.38 | 0.5 | -0.12 |
|  |  |  |  |  |  |  |  |
| Pro | CCG | 0 | 3 | -3 | 0 | 0.5 | -0.5 |
| Pro | CCA | 4 | 1 | 3 | 0.67 | 0.17 | 0.5 |
| Pro | CCT | 2 | 1 | 1 | 0.33 | 0.17 | 0.16 |
| Pro | CCC | 0 | 1 | -1 | 0 | 0.17 | -0.17 |
|  |  |  |  |  |  |  | 0 |
| Gln | CAG | 2 | 3 | -1 | 0.4 | 0.6 | -0.2 |
| Gln | CAA | 3 | 2 | 1 | 0.6 | 0.4 | 0.2 |
|  |  |  |  |  |  |  |  |
| Arg | AGG | 0 | 0 | 0 | 0 | 0 | 0 |
| Arg | AGA | 5 | 0 | 5 | 0.63 | 0 | 0.63 |
| Arg | CGG | 1 | 1 | 0 | 0.13 | 0.13 | 0 |
| Arg | CGA | 0 | 0 | 0 | 0 | 0 | 0 |
| Arg | CGT | 2 | 3 | -1 | 0.25 | 0.38 | -0.13 |
| Arg | CGC | 0 | 4 | -4 | 0 | 0.5 | -0.5 |
|  |  |  |  |  |  |  |  |
| Ser | AGT | 3 | 2 | 1 | 0.23 | 0.15 | 0.08 |
| Ser | AGC | 0 | 4 | -4 | 0 | 0.31 | -0.31 |
| Ser | TCG | 0 | 2 | -2 | 0 | 0.15 | -0.15 |
| Ser | TCA | 2 | 1 | 1 | 0.15 | 0.08 | 0.07 |
| Ser | TCT | 6 | 2 | 4 | 0.46 | 0.15 | 0.31 |
| Ser | TCC | 2 | 2 | 0 | 0.15 | 0.15 | 0 |
|  |  |  |  |  |  |  |  |
| Thr | ACG | 1 | 1 | 0 | 0.2 | 0.2 | 0 |
| Thr | ACA | 3 | 1 | 2 | 0.6 | 0.2 | 0.4 |
| Thr | ACT | 1 | 1 | 0 | 0.2 | 0.2 | 0 |
| Thr | ACC | 0 | 2 | -2 | 0 | 0.4 | -0.4 |
|  |  |  |  |  |  |  |  |
| Val | GTG | 1 | 4 | -3 | 0.1 | 0.4 | -0.3 |
| Val | GTA | 1 | 2 | -1 | 0.1 | 0.2 | -0.1 |
| Val | GTT | 4 | 2 | 2 | 0.4 | 0.2 | 0.2 |
| Val | GTC | 4 | 2 | 2 | 0.4 | 0.2 | 0.2 |
|  |  |  |  |  |  |  |  |
| Trp | TGG | 0 | 0 | 0 | 0 | 0 | 0 |
|  |  |  |  |  |  |  |  |
| Tyr | TAT | 4 | 2 | 2 | 1 | 0.5 | 0.5 |
| Tyr | TAC | 0 | 2 | -2 | 0 | 0.5 | -0.5 |
|  |  |  |  |  |  |  |  |
| End | TGA | 0 | 0 | 0 | 0 | 0 | 0 |
| End | TAG | 1 | 0 | 1 | 1 | 0 | 1 |
| End | TAA | 0 | 1 | -1 | 0 | 1 | -1 |
